# Secretomic analyses of *Ruminiclostridium papyrosolvens* reveal its enzymatic basis for lignocellulose degradation

**DOI:** 10.1186/s13068-019-1522-8

**Published:** 2019-07-15

**Authors:** Zhenxing Ren, Wuxin You, Shasha Wu, Ansgar Poetsch, Chenggang Xu

**Affiliations:** 1 Key Laboratory of Chemical Biology and Molecular Engineering of Ministry of Education, Institute of Biotechnology, Shanxi University, Taiyuan, 030006 Shanxi China; 2 Institute of Applied Chemistry, Shanxi University, Taiyuan, 030006 Shanxi China; 3 Department of Plant Biochemistry, Ruhr University Bochum, 44801 Bochum, Germany; 4 School of Biomedical and Healthcare Sciences, University of Plymouth, Plymouth, PL48AA UK

**Keywords:** *Ruminiclostridium papyrosolvens*, Secretome, Carbohydrate-active enzymes (CAZymes), Cellulosome, ATP-binding cassette (ABC) transporter

## Abstract

**Background:**

Efficient biotechnological conversion of lignocellulosic biomass to valuable products, such as transportation biofuels, is ecologically attractive, yet requires substantially improved mechanistic understanding and optimization to become economically feasible. Cellulolytic clostridia, such as *Ruminiclostridium papyrosolvens* (previously *Clostridium papyrosolvens*), produce a wide variety of carbohydrate-active enzymes (CAZymes) including extracellular multienzyme complexes—cellulosomes with different specificities for enhanced cellulosic biomass degradation. Identification of the secretory components, especially CAZymes, during bacterial growth on lignocellulose and their influence on bacterial catalytic capabilities provide insight into construction of potent cellulase systems of cell factories tuned or optimized for the targeted substrate by matching the type and abundance of enzymes and corresponding transporters.

**Results:**

In this study, we firstly predicted a total of 174 putative CAZymes from the genome of *R. papyrosolvens*, including 74 cellulosomal components. To explore profile of secreted proteins involved in lignocellulose degradation, we compared the secretomes of *R. papyrosolvens* grown on different substrates using label-free quantitative proteomics. CAZymes, extracellular solute-binding proteins (SBPs) of transport systems and proteins involved in spore formation were enriched in the secretome of corn stover for lignocellulose degradation. Furthermore, compared with free CAZymes, complex CAZymes (cellulosomal components) had larger fluctuations in variety and abundance of enzymes among four carbon sources. In particular, cellulosomal proteins encoded by the *cip*-*cel* operon and the *xyl*-*doc* gene cluster had the highest abundance with corn stover as substrate. Analysis of differential expression of CAZymes revealed a substrate-dependent secretion pattern of CAZymes, which was consistent with their catalytic activity from each secretome determined on different cellulosic substrates. The results suggest that the expression of CAZymes is regulated by the type of substrate in the growth medium.

**Conclusions:**

In the present study, our results demonstrated the complexity of the lignocellulose degradation systems of *R. papyrosolvens* and showed the potency of its biomass degradation activity. Differential proteomic analyses and activity assays of CAZymes secreted by *R. papyrosolvens* suggested a distinct environment-sensing strategy for cellulose utilization in which *R. papyrosolvens* modulated the composition of the CAZymes, especially cellulosome, according to the degradation state of its natural substrate.

**Electronic supplementary material:**

The online version of this article (10.1186/s13068-019-1522-8) contains supplementary material, which is available to authorized users.

## Background

Lignocellulosic biomass is the most abundant biopolymer on earth, yet its recalcitrance to hydrolysis has severely hampered its exploitation for renewable energy and materials [1–3]. In nature, direct hydrolysis of lignocellulose is carried out exclusively by enzymes secreted by microorganisms. Enzymes involved in the degradation of these polysaccharides are designated carbohydrate-active enzymes (CAZymes) and classified into five distinct groups according to their activities and structural features: glycoside hydrolases (GHs), polysaccharide lyases (PLs), carbohydrate esterases (CEs), glycosyl transferases (GTs) and enzymes with auxiliary activities (AAs), which often display a modular structure with non-catalytic carbohydrate-binding modules (CBMs). The CAZymes classification system has been integrated and meticulously updated in the CAZy database (http://www.cazy.org) [4, 5].

Cellulolytic clostridia, which are ubiquitous in cellulosic anaerobic environments, represent a major class for efficient biological degradation of cellulosic biomass [6, 7]. Their cellulolytic machinery relies both on cellulosome complexes and on non-cellulosomal free CAZymes [3]. Cellulosome complexes are extracellular multienzyme machineries produced by numerous anaerobic and cellulolytic microorganisms, which consist of a non-catalytic multi-functional integrating subunit (called scaffoldin), responsible for organizing the various catalytic subunits into the complex [8, 9]. The integration is accomplished by the interaction of two complementary module classes, i.e., a cohesin module on the scaffoldin and a dockerin module on each enzymatic subunit [10]. These specific characteristics allow the cellulosome to degrade cellulosic substrates effectively. The host cells and their substrate degradation machineries [11–13] are being exploited in the production of cellulosic biofuels by a variety of approaches, notably consolidated bioprocessing (CBP; [14]).

As an important model for mesophilic anaerobic cellulolytic bacteria, for *Ruminiclostridium cellulolyticum* (previously *Clostridium cellulolyticum*) cellulolytic machinery expression and regulation of metabolism on cellulose and its derivatives have been widely investigated by employing transcriptomics [15, 16] and proteomics [17, 18]. It is known that the expression of CAZymes, including proteins of the cellulosome, is regulated by the type of substrate [15, 17]. However, *Ruminiclostridium papyrosolvens*, essentially the most derived species among known mesophilic cellulolytic clostridia [16], has not yet been studied systematically because of a previous lack of genetic information and transformation method. Recently, genome sequences of two strains (DSM 2782 and C7) of *R. papyrosolvens* have been published [19, 20] and its transformation method has been successfully developed [21], which opens the door to thorough research of *R. papyrosolvens*.

In the present study, to achieve global insight into the cellulolytic machinery of *R. papyrosolvens* DSM 2782, we cultivated *R. papyrosolvens* on four different sources of carbon: glucose, cellobiose, microcrystalline cellulose and corn stover. Thereupon, extracellular proteomes (secretomes) were quantitatively compared by label-free liquid chromatography–tandem mass spectrometry (LC–MS/MS), and their catalytic activities for degradation of different substrates were examined. The results demonstrated that *R. papyrosolvens* modulates composition and abundance of its extracellular enzymes and transporters according to the growth substrate.

## Results

### Genomic features of cellulose degradation for *R. papyrosolvens*

The draft genome of *R. papyrosolvens* DSM 2782 consists of 31 contigs with a GC content of 37.0% and a total length of 4,915,287 bp. It encodes 4039 proteins, 57 tRNAs and 18 rRNAs; 100-mL cultures grown on various carbon sources were harvested when the concentration of extracellular proteins reached the maximum (Additional file 1: Figure S1). After centrifuged (12,000*g*, 4 °C, 30 min), the supernatants were aspirated and filtered through a 0.22-µm PES membrane (Jinteng, Tianjin, China). The residues after centrifugation of the cultures were washed and centrifuged for three times with 5 mL EDTA buffer (50 mM Tris–HCl, 5 mM EDTA, pH8.0), with eluates collected to obtain the proteins binding to the cellulose materials. Cell-free supernatants and the filtered eluates were pooled together and concentrated 100-fold using an ultrafiltration device containing a 10-kDa-cutoff membrane (Millipore, Germany). Protein concentrations were measured by the BCA assay (Sangon Biotech, Shanghai, China). The isolated samples were boiled for 5 min at 100 °C and loaded onto 12.5% SDS-PAGE. Proteins were visualized with a Coomassie Brilliant Blue (CBB-G250) stain as described by Dyballa and Metzger [52] (Additional file 2: Table S1; GenBank Accession Number NZ_ACXX00000000.2; [19]). CAZymes were predicted by HMMER3.0 (http://hmmer.org/) [22] according to dbCAN database [23] definition: a total of 174 CAZyme genes were annotated for *R. papyrosolvens* DSM 2782 genome, including 106 GHs, 3 PLs, 34 CEs and 64 CBM-harboring proteins, in which more than 65% of genes are orthologous to *R. cellulolyticum* (Additional file 3: Table S2). Furthermore, among these CAZyme genes, there are 74 putative cellulosomal subunit-encoding genes, including 71 dockerin-encoding genes and 3 cohesin-encoding genes, which amounts to more than the 65 cellulosomal subunits of *R. cellulolyticum* [15, 17]. However, among them 57 orthologous cellulosomal genes are shared by *R. papyrosolvens* and *R. cellulolyticum* (Additional file 3: Table S2). Thus, it is suggested that *R. papyrosolvens* has evolved a very sophisticated cellulolytic system, which has remarkable orthologous relationships with *R. cellulolyticum* [24].

**Fig. 1:**
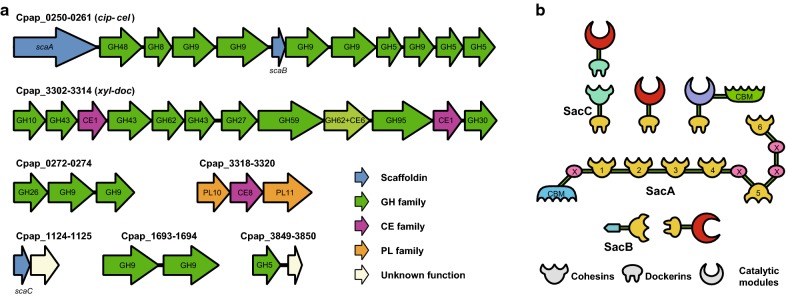
Cellulosome system of *Ruminiclostridium papyrosolvens*. **a** Cellulosome-related gene clusters in the genome of *R. papyrosolvens*. **b** Schematic representation of architecture of the *R. papyrosolvens* cellulosome

Cellulosomal genes from mesophilic clostridia tend to physically cluster along the chromosome [8], and *R. papyrosolvens* is no exception. Among the 74 cellulosomal genes in total, we identified seven clusters (Fig. 1a): (i) the “*cip*-*cel*” gene cluster of 12 genes (Cpap_0250-0261) that encodes the major cellulosome components, including two cohesin-harboring scaffoldins, respectively, named ScaA and ScaB. SacA is composed of six cohesin domains of type I numbered from 1 to 6 from the N- to the C-terminus, in addition to a N-terminal cellulose-binding module (CBM) and four X modules, separated by short linker sequences, while SacB only harbors a cohesin domain in its C-terminus (Fig. 1b); (ii) the “*xyl*-*doc*” cluster of 12 genes (Cpap_3302-3314) encoding exclusively secreted dockerin-containing proteins, which are probably involved in hemicellulose degradation and herein named the *xyl*-*doc* gene cluster [17]; (iii) a couple of genes arranging another cohesin–dockerin interaction (named type II to distinguish from primary cohesin–dockerin interaction of type I, Cpap_1124-1125), in which Cpap_1124 encodes a type II cohesin domain along with the type I dockerin (named *scaC*), while Cpap_1125 encodes a type II dockerin, resulting that it is assembled into Cpap_1124-encoded type II cohesin, and then attached to the primary scaffoldin by mediation of type I dockerin of Cpap_1124 (Fig. 1b); (iv) other small clusters (two or three genes) encoding cellulosomal enzymes (Cpap_0272-0274, Cpap_1693-1694, Cpap_3318-3320 and Cpap_3849-3850), in which two clusters, (Cpap_0272-0274 and Cpap_3318-3320), respectively, are located downstream of the *cip*-*cel* and *xyl*-*doc* clusters.

### Structure of secretome profiles in *R. papyrosolvens*

To identify the components of the cellulose degradation in *R. papyrosolvens*, we started by characterizing the populations of extracellular proteins in *R. papyrosolvens* cultures under a variety of carbon sources using proteomics with label-free quantitation. The carbohydrate substrates tested included cellulose and its derivatives glucose and cellobiose, and corn stover, the natural plant-derived lignocellulose. Growth was assessed by monitoring protein concentration of fermentation supernatants (Additional file 1: Figure S1). Proteins secreted to fermentation supernatants were concentrated by ultrafiltration when their concentrations reached the maximum and then analyzed by SDS-PAGE (Additional file 4: Figure S2). Total extracellular proteins isolated from each of the four growth conditions in three biological replicates were analyzed by label-free LC–MS/MS. Proteins were identified using *R. papyrosolvens* protein sequence data from NCBI. In total, 1151 Protein sequences were found in the secretome (i.e., around 28% of the total 4172 proteins encoded in *R. papyrosolvens* genome).

**Fig. 2:**
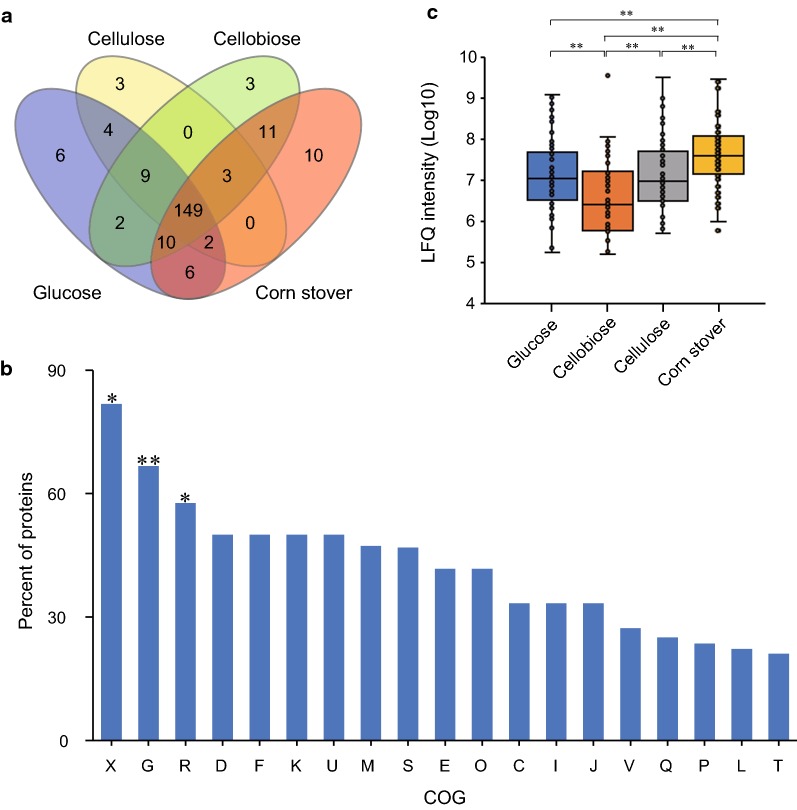
Label-free LC–MS/MS analysis of *R. papyrosolvens* secretomes. **a** Venn diagram of total protein numbers for *R. papyrosolvens* secretomes in glucose, cellobiose, cellulose, and corn stover media. **b** Functional profile of proteins identified in secretomes. Percentages of such proteins in each COG term were shown in columns (X, mobilome: prophages, transposons; G, carbohydrate transport and metabolism; R, general function prediction only; D, cell cycle control, cell division, chromosome partitioning; F, nucleotide transport and metabolism; K, transcription; U, intracellular trafficking, secretion, and vesicular transport; M, cell wall/membrane/envelope biogenesis; S, function unknown; E, amino acid transport and metabolism; O, posttranslational modification, protein turnover, chaperones; C, energy production and conversion; I, lipid transport and metabolism; J, translation, ribosomal structure and biogenesis; V, defense mechanisms; Q, secondary metabolites biosynthesis, transport and catabolism; P, inorganic ion transport and metabolism; L, replication, recombination and repair; T, signal transduction mechanisms). Proteins identified were enriched in G, X and R of COG categories (*P < 0.05, **P < 0.01, hypergeometric test). **c** Box plot of differential expression of COG category of G in four secretomes under four carbon sources (*P < 0.05, **P < 0.01, *t*-test)

Proteomes were obtained for three biological replicates under each carbon source. The results of PCA analysis indicated that the respective three biological replicates always closely cluster in the first and second components. However, there are much bigger distances between samples from different substrates, suggesting that the secretomes were significantly distinguished by their substrates (Additional file 5: Figure S3). In this study, a reliably expressed protein was defined as being identified in at least two biological replicates for one certain carbon source. Based on this principle, 912 proteins in total were found to be expressed in our experiments, of which 218 proteins were predicted to harbor signal peptides using SignalP5.0 [25] (Additional file 6: Table S3). Among them, 188, 170, 187 and 191 proteins were, respectively, identified under glucose, cellobiose, cellulose and corn stover; 149 proteins were shared among all four conditions being the core components of secretome, whereas the 22 exclusive proteins are most likely linked to the respective substrate (Fig. 2a, Additional file 7: Table S4).

To probe the functional implication of secretomes, 218 proteins with signal peptides were examined for association with functional categories as defined by Cluster of Orthologous Group (COG [ftp.ncbi.nih.gov/pub/COG/COG2014/static/lists/homeCOGs.html]; Additional file 7: Table S4). A broad spectrum of COGs was found, with these proteins being enriched in COG categories such as G (carbohydrate transport and metabolism), X (Mobilome: prophages, transposons) and R (General function prediction only) (*P*-value < 0.05, hypergeometric test; Fig. 2b). Furthermore, expression of proteins in COG category of G with the highest degree of enrichment was compared among four secretomes (Fig. 2c). The box plot showed that expression of proteins in G category was significantly different between any two substrates except glucose and cellulose, and among them, expression between cellobiose and corn stover produced the highest difference (Fig. 2c). Thus, it is suggested that *R. papyrosolvens* secretes different proteins related to carbohydrate transport and metabolism according to its growth substrates. Interestingly, expression pattern of these proteins in glucose appears to be similar to cellulose.

**Fig. 3:**
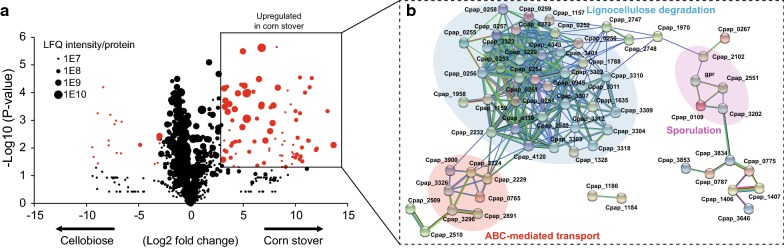
Expression difference between cellobiose and corn stover secretomes of *R. papyrosolvens* secretomes. **a** Protein abundance in secretome for corn stover vs cellobiose. Log2-transformed fold change was plotted against log10-transformed *P*-value (*t*-test). Red-colored dots indicate proteins with log2 > 3 and *P* < 0.05. **b** A network analysis was performed on proteins highlighted in red in (**a**) according to protein–protein interactions using STRING database [26]. The formed protein clusters were differentially colored, and functional definitions were provided according to clusters

We then compared secretomic profiles between cellobiose and corn stover, which are, respectively, considered as easily and difficultly consumed carbon sources. It is observed that there are more high-abundant, up-regulated proteins under corn stover in comparison with cellobiose (Fig. 3a). To determine the clustering function of these up-regulated proteins under corn stover, we applied a network analysis using STRING [26]. The result showed that up-regulated proteins displayed functionally distinct clusters. Compared with cellobiose, *R. papyrosolvens* consuming corn stover had increased expression of CAZymes including cellulosomal components for lignocellulose degradation, extracellular SBPs of ATP-binding cassette (ABC) transporters and proteins involved in spore formation (Fig. 3b). We concluded that this proteomic evidence was consistent with corn stover exhibiting a complex structure composed of cellulose, hemicellulose, lignin and other soluble sugars. For utilization of corn stover, cells need to secrete an arsenal of degradative CAZymes and ABC transporters. Meanwhile, corn stover can be regarded as adverse environmental condition compared with cellobiose, triggering expression of proteins involved in sporulation in *R. papyrosolvens*. The similar phenomenon had been reported in *R. thermocellum* and *R. cellulolyticum*. Attachment to cellulose fibers could trigger sporulation in *R. thermocellum* [27], while Spo0A mutant of *R. cellulolyticum* abolished the sporulation ability and increased dramatically cellulose catabolism [28].

### Expression pattern of CAZymes

To further probe the links among the substrate-specific proteins being implicated in plant cell wall degradation, we firstly counted the types and numbers of expressed CAZymes under the different substrates. In total, 116 of 174 CAZymes encoded by *R. papyrosolvens* genome were retrieved in its secretome. These expressed CAZymes were mainly distributed in COG categories of G, R and M. In particular, more than half (70) of CAZymes belonged to G category, which accounted for 51% of all proteins in this category. Furthermore, these secreted CAZymes included 67 cellulosomal subunits and 49 non-cellulosomal CAZymes, respectively, accounting for 91% and 49% of their genome-predicted proteins. Moreover, the abundance of non-cellulosomal CAZymes and cellulosomal subunits, respectively, accounted for 2.4% and 10.1% of the total abundance of secretome.

**Fig. 4:**
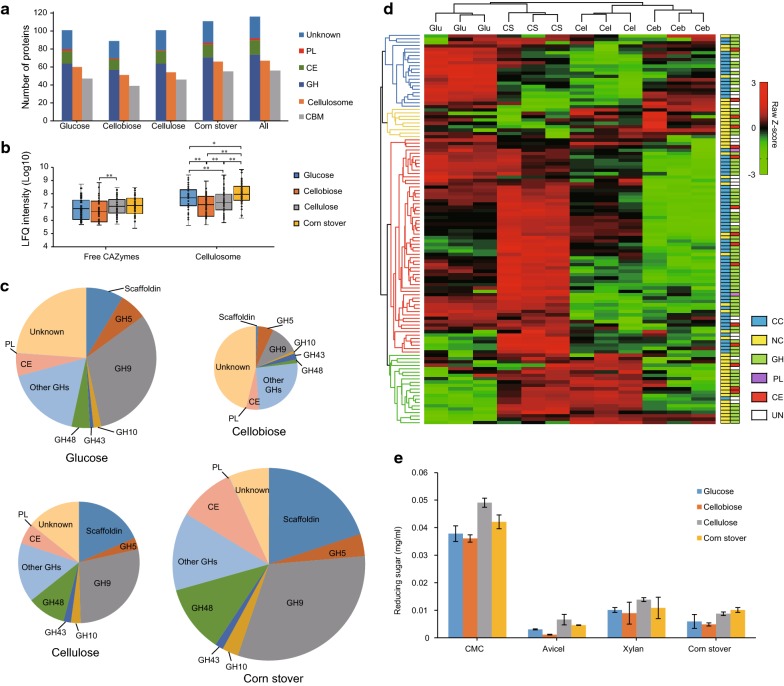
Comparison of expression and activity of CAZymes from four secretomes. **a** Functional and structural classification of the CAZymes released by *R. papyrosolvens* grown on glucose, cellobiose, cellulose and corn stover. **b** Distribution of abundance of cellulosomal CAZymes and free CAZymes in various conditions (*P < 0.05, **P < 0.01, *t*-test). **c** Proportion of major CAZyme families in total expressed CAZymes under four culture conditions. **d** Expression profiles of CAZymes under the selected carbon sources were clustered into four groups by hierarchical clustering analysis. The structural [cellulosomal component (CC) and non-cellulosomal enzyme (NC)] and functional [glycoside hydrolase (GH), glycosyltransferase (GT), carbohydrate esterase (CE), polysaccharide lyase (PL) and unknown function enzyme (UN)] characteristics of CAZymes were distinguished by different color blocks. **e** Comparative hydrolysis of cellulosic CMC and Avicel, xylan and corn stover by enzymes in the secretomes of *R. papyrosolvens* grown on glucose, cellobiose, cellulose and corn stover. The values shown are the means of three replicates, and the error bars indicate standard deviations from the mean values (*P < 0.05, **P < 0.01, *t*-test)

Out of the in total 116 expressed CAZymes, 101, 89, 101 and 111 CAZymes were, respectively, identified under glucose, cellobiose, cellulose and corn stover, demonstrating that cells secreted the most CAZymes, cellulosomal components and CBM-harboring proteins when grown on corn stover (Fig. 4a). Interestingly, *R. papyrosolvens* expressed more CAZymes, cellulosomal components and CBM-harboring proteins under glucose than cellobiose. Meanwhile, results of growth curves indicated that *R. papyrosolvens* preferred cellobiose over glucose (Additional file 1: Figure S1). These results on *R. papyrosolvens* are completely consistent with our previous findings on *R. cellulolyticum*, suggesting activation of cellulase expression by non-preferred carbon source (i.e., glucose) and inhibition by a preferred substrate (i.e., cellobiose), which can be explained by the carbon catabolite repression (CCR) mechanism [15]. Furthermore, qualitative and quantitative differences of CAZymes among four carbon sources were mainly from cellulolosomal components. For example, the number of cellulolosomal components increased from 51 in cellobiose to 66 in corn stover. CAZymes compared between cellulose and corn stover comprised nearly equal numbers of GH, CE and GT family enzymes (Fig. 4a). On the other hand, the expression of free CAZymes had no difference among four substrates (except between cellobiose and cellulose) with nearly equal average abundances, but expression of cellulosomal CAZymes was significantly different between any two substrates and the average abundances of cellulosomal subunits had the same tendency of changes with its number among four carbon sources, i.e., the cellulosome had the highest relative abundance under corn stover and the lowest expression abundance under cellobiose (Fig. 4b). This indicated that additional cellulosomal components with high expression level are required for degradation of the more structurally complex substrate lignocellulose.

It has been proved that scaffoldin, GH48 and GH9 family enzymes are the most abundant components of cellulosome, and GH5, GH10, GH43, CE and PL family enzymes are responsible for degradation of hemicellulose [9, 17]. Next, we investigated the proportion of these major CAZymes in total CAZymes under various carbon sources (Fig. 4c). The results indicated the largest portion of CAZymes in secretome was from corn stover, but the smallest was from cellobiose among four carbon sources. On the other hand, the proportion of key cellulosomal subunits (scaffoldin, GH48 and GH9 family proteins) and CE family proteins significantly increased with increasing proportion of CAZymes in secretome. For example, the proportion of scaffoldin increased from 1% in cellobiose to 20% in corn stover, while that of proteins with unknown function decreased from 46 to 7%. However, the proportion of GH5, GH10 and GH43 family proteins had no difference under four culture conditions. Thus, we proposed that these major cellulosomal subunits, especially scaffoldin, GH48 and GH9 family, were the core components of degradation system of lignocellulose, and CE and GH5, GH10 and GH43 family proteins also play important roles in hydrolysis of plant wall polysaccharides.

Based on their substrate-dependent expression patterns, the 116 CAZymes were clustered into four different groups (Fig. 4d, Additional file 8: Table S5). Group 1 (blue) included 21 CAZymes that showed the highest relative abundance under glucose, which belong to GH families and proteins of unknown function harboring a CBM or dockerin domain. Most of this group of CAZymes were also cellulosomal components, of which 15 proteins harbor the dockerin domain. Group 2 (orange) included 9 CAZymes that showed higher relative abundance under cellobiose and cellulose than glucose and corn stover. CAZymes of this group were free CAZymes. Group 3 (red) was the largest group including 64 CAZymes that showed the highest relative abundance under corn stover and the lowest relative abundance under cellobiose. CAZymes of this group were mainly cellulosomal components, of which 50 proteins harbor dockerin or cohesin domains. Surprisingly, most of the cellulosomal subunits encoded by the *cip*-*cel* and *xyl*-*doc* gene clusters belonged to this group, suggesting that cellulosomal CAZymes, especially the key subunits encoded by the two large gene clusters, were the primary degraders of lignocellulose, such as corn stover. Group 4 (green) included 22 proteins that showed higher relative abundance under cellulose and corn stover than soluble sugars glucose and cellobiose. Proteins of this group were mostly free CAZymes. Thus, we found clear distinction on expression pattern between free CAZymes and cellulosomal components, suggesting that they could be controlled by different and likely independent mechanisms. Furthermore, the expressions of all the 116 CAZymes demonstrated a negative correlation between cellobiose and corn stover, with a high correlation coefficient (*R *= 0.67) (Additional file 9: Figure S4). Thus, it is suggested that the more difficult the substrate to be utilized, the more types and abundances of CAZymes are secreted by the bacterium.

To test the correlation between expression of CAZymes and their catalytic activity, we examined these four isolated extracellular samples for their degradation of four polysaccharide substrates: carboxymethyl cellulose (CMC), microcrystalline cellulose (Avicel), xylan and corn stover by the measurement of released total reducing sugars using the DNS method [29] (Fig. 4e). The results indicated that samples isolated from cellulose and corn stover were more efficient on all polysaccharide substrates, respectively, while samples from cellobiose had the lowest activities. Samples from cellulose were most efficient to hydrolyze CMC and Avicel, and the most active samples on corn stover were samples isolated from corn stover. Thus, the catalytic activity of CAZymes has a strong link to supplied substrate, in which cells produce the optimal formulation of CAZymes.

### Expression of key CAZymes and ABC transporters

**Fig. 5:**
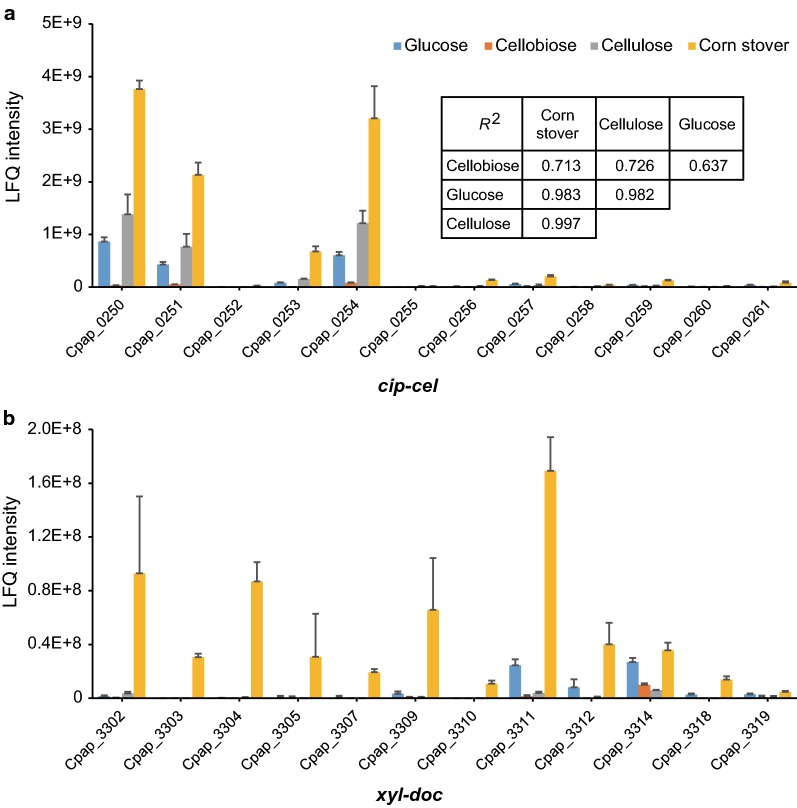
Expression of *cip*-*cel* (**a**) and *xyl*-*doc* (**b**) cellulosomal gene clusters. The expression correlation of the 12 *cip*-*cel* genes under various carbon sources was calculated and compared. The values shown are the means of three replicates, and the error bars indicate standard deviations from the mean values

We first compared expression of cellulosomal subunits encoded by both of the *cip*-*cel* and *xyl*-*doc* clusters among different carbon sources (Fig. 5). The proteomic data reveal that expression of the *cip*-*cel* cluster appears to be negatively correlated with availability or preference of carbon sources due to the order of the average expression level of *cip*-*cel* (corn stover > cellulose > glucose > cellobiose). On the other hand, 12 proteins of *cip*-*cel* exhibit highly uneven abundance under all the four carbon sources tested, where the observed relative abundance of the first (Cpap_0250, encoding the scaffoldin ScaA), second (Cpap_0251, encoding an exoglucanase of GH48) and fifth (Cpap_0254, encoding an endoglucanase of GH9) genes was far higher than the other genes in the cluster (Fig. 5a). Interestingly, the ratio among 12 proteins of *cip*-*cel* was independent of the carbon sources, as demonstrated by the high correlations of abundance of *cip*-*cel* proteins among four carbon sources (*R*^2^ ranged from 0.637 to 0.997) (Fig. 5a). These results of *cip*-*cel* in *R. papyrosolvens* are in remarkably agreement with those in *R. cellulolyticum* in which expression difference of *cip*-*cel* was caused by the mechanism of selective RNA processing and stabilization (SRPS) in post-transcriptional level [15, 16], suggesting that the *cip*-*cel* cluster is not only conserved in protein-coding sequences, but also in regulation mechanism between *R. papyrosolvens* and *R. cellulolyticum*.

Like the *cip*-*cel* cluster, the *xyl*-*doc* cluster had the highest relative abundance under corn stover with highly uneven abundance but was hardly expressed under other three carbon sources, except that the last five genes were expressed under glucose (Fig. 5b). It is fully consistent with our previous study on analysis of the promoter activity upstream of *xyl*-*doc* [21] and other group’s study on transcription analysis of *xyl*-*doc* from *R. cellulolyticum* [30]. In short, the *cip*-*cel* and *xyl*-*doc* clusters from *C. papyrosolvens* were expressed in a manner very similar to that from *R. cellulolyticum*, implying that they may be regulated by many mechanisms that had been proved in *R. cellulolyticum*, such as CCR [15, 31], two-component system (TCS) [15, 30, 32] and SRPS [16].

**Fig. 6:**
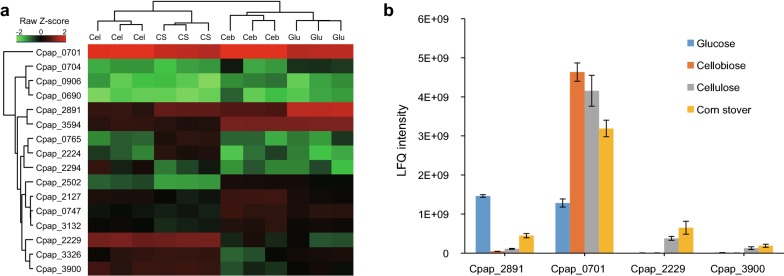
Expression of extracellular subunits of ABC transporters SBPs in four secretomes of *R. papyrosolvens*. **a** Hierarchical clustering of the 16 expressed SBPs under glucose, cellobiose, cellulose and corn stover. **b** Expression comparison of four typical CBPs (Cpap_0701, Cpap_2229, Cpap_2891 and Cpap_3900) with the highest protein abundance among all SBPs in secretomes. The values shown are the means of three replicates, and the error bars indicate standard deviations from the mean values

In addition to CAZymes for degradation of lignocelluloses, bacteria are required to employ sugar transporters to transport lignocellulosic degradation products into cells (Fig. 3b). Thus, we further analyzed the expression of SBPs (extracellular subunits of ABC transporters) from secretome. In total 16 SBPs were found in our secretome, which was categorized into groups based on abundance trend similarity (Fig. 6a). It shows that some SBPs (Cpap_0690, Cpap_0704 and Cpap_0906) were expressed at extremely low level among all substrates, while Cpap_0701 had high relative abundance in all substrates. And others are expressed differentially among four carbon sources.

We further analyzed the expression pattern of four CBPs (Cpap_0701, Cpap_2229, Cpap_2891 and Cpap_3900) with the highest relative abundance (Fig. 6b). Cpap_2891 had the highest relative abundance on glucose, moderate relative abundance on corn stover and low relative abundance on cellobiose and cellulose, suggesting that Cpap_2891 was potentially in charge of glucose transportation. Both Cpap_2229 and Cpap_3900 had high relative abundance on cellulose and corn stover, and almost no expression on glucose and cellobiose, suggesting that they play an important role in transportation of oligosaccharides.

It is worth noting that Cpap_0701 accounted for the most mass of SBP elements, which made up more than 70% of all SBPs in secretome (Additional file 6: Table S3). It had much higher relative abundance on cellobiose, cellulose and corn stover than glucose (Fig. 6b), with same expression pattern as its ortholog CauA (Ccel_2112, sequence 94% identity) from *R. cellulolyticum*. CauA-harboring ABC transporter in *R. cellulolyticum* is provably devoted to cellobiose and cellodextrins uptake and regulated by its upstream two-component system [15, 32]. This suggests that the ABC transporter harboring Cpap_0701 also facilitates an influx of cellobiose and cellodextrins in *R. papyrosolvens*.

## Discussion

This study explores the complexity of the plant cell wall degradation system of *R. papyrosolvens*. Whole-genome analysis of *R. papyrosolvens* revealed a 174-CAZyme repertoire of 106 GHs, 34 CEs, 3 PLs and 64 CBMs, including 74 cellulosomal components, suggesting diversity and substrate adaptation in enzymatic activity. The most abundant GH families were GH5, GH9 and GH43, which constituted over 50% of the enzymatic domains identified. Our results were slightly different from the previous CAZyme prediction in *R. papyrosolvens* [24], in which there are 127 CAZymes including 103 GHs, 19 CEs, 5 PLs, 67 CBMs and 70 cellulosomal components. This may be due to the different analytical methods and versions of genome annotation. However, both of CAZyme predictions in *R. papyrosolvens* revealed that CAZymes of *R. papyrosolvens* represented a notable increase compared to CAZymes observed in *R. cellulolyticum* [19, 24]. In addition to 115 orthologs of *R. cellulolyticum*, *R. papyrosolvens* evolved more particular enzymes to degrade unusual polysaccharides to adapt to the complex environment, such as GH31, GH39, GH109 and GT and CE families. The differences in numbers may be attributed to the size of the genomes, which is 4.92 Mb for *R. papyrosolvens* and 4.07 Mb for *R*. *cellulolyticum* [24].

Cellulosome architectures vary greatly among the cellulosome-producing bacterial species, but two global types of architectures of cellulosome systems have been observed, namely simple and complex [8]. Simple cellulosomes have so far been observed in mesophilic clostridial species, such as *Ruminiclostridium cellulovorans* [33], *R. cellulolyticum* and *R. josui* [34]. The simple cellulosome architecture includes a single scaffoldin protein, encoded by *cip*-*cel* cluster, comprising the primary scaffoldin gene followed downstream by a series of genes encoding for various dockerin-bearing enzymes. On the other hand, complex cellulosome systems contain multiple scaffoldin proteins. The major scaffoldin genes are clustered in the genome in a gene cluster. Complex cellulosomes have been observed in *Ruminiclostridium thermocellum* [35], *Bacteroides cellulosolvens* [36], *Acetivibrio cellulolyticus* [37] and *Ruminococcus flavefaciens* [38].

In addition to the *cip*-*cel* cluster, it was found that *R. papyrosolvens* harbors another two-gene cluster (Cpap_1124-1125), encoding type II of scaffoldin protein and dockerin, distinguished from primary cohesin–dockerin interaction of *cip*-*cel* (Fig. 1b). Thus, *R. papyrosolvens* does not only possess the classic simple cellulosomes as mesophilic clostridia, but also has started to evolve the type II of cohesin–dockerin interaction-forming complex cellulosomes. Its architecture is constructed by two types of cohesin–dockerin interaction of scaffoldin and enzymes, which are, respectively, encoded in two-gene clusters comprising a scaffoldin gene followed downstream by enzyme genes (Fig. 1a). It is very different from the complex cellulosomes from *R. thermocellum*, in which type II of cohesin–dockerin interaction happens between two scaffoldins, but not between scaffoldins and enzymes, as they do in cellulosomes from *R. papyrosolvens*. The diversity of CAZymes and cellulosome architectures suggests that the various individual mesophilic clostridial species have evolved several specific strategies for carbohydrate degradation, some similar to, but others distinct from those of their intimate relatives.

To identify the particular proteins that were actually produced under specific substrate conditions including soluble glucose and cellobiose and insoluble cellulose and corn stover, the extracellular secretomes were analyzed by label-free quantification (LFQ) proteomic method in MaxQuant, allowing inter- and intra-experiment comparison of relative protein abundances. However, there is a challenge with isolation of secretomes from insoluble substrates. Genome of *R. papyrosolvens* encodes 64 CBM-harboring proteins including cellulosomal scaffoldin (Additional file 3: Table S2), which potentially bind to insoluble polysaccharides via their CBM. Thus, cellulases harboring CBM might be underestimated in the secretomes, if they are not recovered from their insoluble substrates prior to quantitative proteomics. To improve recovery of these proteins, two alternative strategies may be adopted: one is that samples are collected after exhaustion of insoluble substrates, as binding proteins would be released then. However, such nutrient-depletion stress should affect the secreted proteome—in the worst case intracellular proteins are released due to cell death and confound results. This has been described for secretomes from bacteria at stationary growth phase (e.g., Indrelid et al. [39]). Thus, another approach is concentrating culture supernatants from insoluble substrates by ideally quantitative washes/extraction. The latter procedure has been successfully adopted in many cellulolytic microorganisms [40–43]. On the other hand, studying structure of a family 3 CBM from the cellulosomal scaffoldin subunit of *R. thermocellum* revealed that it harbors a calcium binding loop, whose interaction with Ca^2+^ modulates the mechanostability of CBM [44, 45]. Thus, in order to ensure the completeness of our secretomes, we washed the residual cellulose materials using EDTA buffer to chelate Ca^2+^, thus releasing cellulose-binding proteins as much as possible. We estimated the elution effect of EDTA buffer for cellulose-binding proteins. The results indicated that EDTA buffer was able to elute protein from cellulose effectively, except very few individual proteins (Additional file 10: Figure S5). According to quantitative image analysis of the stained gel, this approach recovered 99% of the secreted proteins. Therefore, the method employed here avoided secretome interference from dying cells while providing quantitative recovery for the majority of insoluble substrate-bound proteins. Admittedly, incomplete removal of individual proteins from insoluble substrates could adversely affect their quantitative comparison with soluble substrates.

In this study, in total 917 proteins were identified in four different sets of secretomes isolated from *R. papyrosolvens* and the LFQ intensity ranged from 4.65 × 10^5^ to 4.56 × 10^9^. Compared to proteomic data of *R. cellulolyticum* [17] and *R. thermocellum* [46], our experiments detected more cellulosomal proteins in more complex samples in which cellulosomes were not purified and enriched. The LFQ protein data revealed that many extracellular proteins, and especially CAZymes, were differentially expressed among various carbon sources. As expected, *R. papyrosolvens* like many cellulolytic bacteria, such as *R. cellulolyticum* [15, 17] and *R. thermocellum* [46], expressed 27% more CAZymes on recalcitrant substrate corn stover than on its preferred sugar cellobiose. However, *R. papyrosolvens* expressed more CAZymes on glucose than cellobiose, being consistent with our previous transcriptomic data for *R. cellulolyticum* [15]. Whereas *R. acetobutylicum* [47] and *R. cellulovorans* [48], the mesophilic phylogenetic relatives of *R. papyrosolvens*, prefer glucose; in the latter, cellulases were transcriptionally repressed under glucose, but derepressed upon glucose exhaustion [48]. Thus, cellulolytic bacteria seem to activate expression of CAZyme by the non-preferred carbon sources and inhibit expression in the preferred substrates, a fact that can be explained by the carbon catabolic repression mechanism (CCR) [15]. Furthermore, cellobiose as the preferred sugar of cellulolytic bacteria, such as *R. papyrosolvens*, *R. cellulolyticum* and *R. thermocellum*, has two advantages: One is avoiding direct diet competition of cellulolytic organisms (who are often in minority, e.g., in the rumen only ~ 10% of the bacteria are cellulolytic [49]) with non-cellulolytic bacteria in carbon source. For most heterotrophic bacteria studied to date, glucose is the preferred (or primary) carbon source [50]. The other is that uptake of cellobiose or cellodextrins into the cell is more energy efficient than glucose. It is because the breakdown of cellobiose and cellodextrins into glucose-1-phosphate by the intracellular cellobiose/cellodextrin phosphorylase does not require the extra ATP [51].

## Conclusions

In this study, secretomes of *R. papyrosolvens* under various carbon sources (glucose, cellobiose, cellulose and corn stover) were investigated and compared by label-free LC–MS/MS quantification method; 912 proteins in total were found in four secretomes which, respectively, harbored a different functional profile of proteins. Compared with cellobiose, secretome under corn stover had increased expression of CAZymes for lignocellulose degradation, extracellular SBP from ATP-binding cassette (ABC) transporters and proteins involved in spore formation. Surprisingly, the number and abundance of free CAZymes were almost unchanged among four carbon sources, whereas the abundance of CAZymes from cellulosome complexes, particularly those encoded by the *cip*-*cel* operon and the *xyl*-*doc* gene cluster, was significantly increased along with their numbers as cells were grown on corn stover. These features do not only delineate the secretome of lignocellulose degradation in *R. papyrosolvens*, but also provide some targets for development of highly efficient biomass degradation systems by employing cellulolytic clostridia and their enzyme system.

## Materials and methods

### Strains and culture conditions

*Ruminiclostridium papyrosolvens* DSM 2782 was purchased from the Leibniz Institute DSMZ (German Collection of Microorganisms and Cell Cultures, Braunschweig, Germany). *R. papyrosolvens* was cultured anaerobically at 35 °C in 250-mL flasks with 100 mL working volume of modified DCB-1 medium supplemented with 2.0 g/L of glucose, cellobiose or 5.0 g/L of cellulose (Avicel PH101; Sigma-Aldrich) or corn stover, which was obtained from Taigu County, Shanxi Province, China, and milled using a beater pulverizer to pass through a mesh with a diameter of 2 mm. A 1% (vol/vol) inoculum of culture preadapted to various substrates in vials was used for inoculation. Cellular growth was measured based on the increase in extracellular proteins in the culture using the bicinchoninic acid (BCA) assay (Sangon Biotech, Shanghai, China). All cultivations were performed in triplicate.

### Isolation of extracellular proteins

100-mL cultures grown on various carbon sources were harvested when the concentration of extracellular proteins reached the maximum (Additional file 1: Figure S1). After centrifuged (12,000*g*, 4 °C, 30 min), the supernatants were aspirated and filtered through a 0.22-µm PES membrane (Jinteng, Tianjin, China). The residues after centrifugation of the cultures were washed and centrifuged for three times with 5-mL EDTA buffer (50 mM Tris–HCl, 5 mM EDTA, pH8.0), with eluates collected to obtain the proteins binding to the cellulose materials. Cell-free supernatants and the filtered eluates were pooled together and concentrated 100-fold using an ultrafiltration device containing a 10-kDa-cutoff membrane (Millipore, Germany). Protein concentrations were measured by the BCA assay (Sangon Biotech, Shanghai, China). The isolated samples were boiled for 5 min at 100 °C and loaded onto 12.5% SDS-PAGE. Proteins were visualized with a Coomassie Brilliant Blue (CBB-G250) stain as described by Dyballa and Metzger [52].

To estimate the elution effect of EDTA buffer for cellulose-binding proteins, 1 mL isolated extracellular proteins from glucose culture (2 mg/mL) were incubated with 0.1 g cellulose (Avicel PH101) for 24 h at 35 °C in vitro and then washed the cellulose 3 times with 1 mL EDTA buffer. Finally, the washing solutions and residual proteins binding to cellulose were analyzed by SDS-PAGE and their amount calculated by gray scanning.

### Label-free quantitative LC–MS/MS analysis

#### Proteolysis

For the proteome assay, samples (50 µg per lane) were allowed to run 2 cm beyond the stacking gel of SDS-PAGE. Protein bands were excised from the gels, cut into small cubes (ca. 1 × 1 mm^3^) and destained according to Schluesener and colleagues [53]. Gel pieces were dried by incubation with 100% acetonitrile for 10 min at room temperature and then incubated with 50 mM DTT in 25 mM NH_4_HCO_3_ (30 min at 60 °C) to reduce proteins disulfide bonds. The gel pieces were dried again with acetonitrile, and proteins were alkylated treating the gel pieces with 50 mM iodoacetamide in 25 mM NH_4_HCO_3_ (1 h at room temperature in darkness). And then gel pieces were dried in a SpeedVac; trypsin (Sequencing grade modified; Promega, Madison, USA) solution (12.5 ng/mL in 25 mM ammonium bicarbonate, pH 8.6) was added until gel pieces were immersed completely in digestion solution (~ 200 µL). Protein digestion was performed overnight at 37 °C with a tempered shaker. After digestion and peptide extraction from gel pieces, the samples were centrifuged, and supernatants were transferred to new 1.5-mL tubes. The recovered peptides were dried using a SpeedVac and stored at room temperature. Prior to MS analysis, peptides were resuspended in 20 µL of 0.1% formic acid. Each measurement was performed with 8 μL of sample.

#### Mass spectrometry analysis

An UPLC HSS T3 column (Waters, Milford, MA, USA) and an UPLC Symmetry C18 trapping column (Waters, Milford, MA, USA) for LC as well as a PicoTip Emitter (SilicaTip, 10 mm i.d., New Objective, Woburn, MA, USA) were used in combination with the nanoACQUITY gradient UPLC pump system (Waters, Milford, MA, USA) coupled to a LTQ Orbitrap Elite mass spectrometer (Thermo Fisher Scientific Inc., Waltham, MA, USA). The peptides were eluted with a 105-min gradient of 2% to 85% acetonitrile with 0.1% formic acid at a flow rate of 400 nL/min (0–5 min: 2%; 5–10 min: 2–5%; 10–71 min: 5–30%; 72–77 min: 85%; 77–105 min: 2%). The LTQ Orbitrap Elite was operated via instrument method files of Xcalibur (Rev. 2.1.0) in positive ion mode. The linear ion trap and Orbitrap were operated in parallel, i.e., during a full MS scan on the Orbitrap in the range of 150–2000 *m*/*z* at a resolution of 240,000 MS/MS spectra of the 20 most intense precursors were detected in the ion trap using the rapid scan mode. The relative collision energy for collision-induced dissociation (CID) was set to 35%. Dynamic exclusion was enabled with a repeat count of 1- and 45-s exclusion duration window. Singly charged and ions of unknown charge state were rejected from MS/MS.

#### Protein identification

Protein identification was performed by Andromeda search engine [54] embedded in MaxQuant searching against the complete proteome database (GCF_000175795.2) of *R. papyrosolvens* DSM2782 according to genome annotation in NCBI database (https://www.ncbi.nlm.nih.gov/). Mass tolerance for centroid match was set to 8 ppm; the mass tolerance for fragment ions was set to 0.4 Da. The PSM false discovery rate (FDR) and protein FDR were determined with MaxQuant, and the *q*-value was set to 1% [55]. For protein quantification, the “label-free quantification” function in MaxQuant was used.

### Functional enrichment analysis

The statistical significance of the enrichment of proteins from the secretomes in each COG category was calculated as follows: For example, let “*N*” be the total number of proteins predicted in all COG categories, “*n*” be the number of proteins predicted in a certain COG category, “*M*” be the total number of proteins identified in secretomes, and “*m*” be the number of secretomic proteins assigned to this COG category. The *P*-value was estimated for enrichment of proteins identified in a COG category based on the hypergeometric test:

$$P = \sum\limits_{i = m}^{\text{min} (n,M)} {\frac{C(M,i)C(N - M,n - m)}{C(N,n)}}$$

in which *C*(*x*,*y*) is the combinational number of choosing *y* items out of *x* items. Enrichment of COG-slim terms with *P*-value ≤ 0.05 was considered as statistically significant.

### Enzyme activity measurement

Activity assays of CAZymes were performed by incubating 0.1 mg/mL isolated secretome samples in a total volume of 500 μL assay mixture containing 1% (wt/vol) of substrates (CMC, Avicel, oat spelt xylan, or milled corn stover) in MES buffer (50 mM MES, 5 mM CaCl_2_, pH6.0) at 50 °C for 8 h. The released sugar concentration was estimated by dinitrosalicylic acid (DNS) method [29] using glucose as standard. The absorbance was measured at 540 nm. All experiments were performed in triplicate.

## Additional files

**Additional file 1: Figure S1.** Growth curves of *Ruminiclostridium papyrosolvens* on glucose, cellobiose, cellulose and corn stover. Cell growth was monitored by determining the amount of proteins in supernatant of fermentation broths as described in Materials and Methods. The symbols indicate the means of three experiments, and the error bars indicate the standard deviations. The arrows show the time of sampling for MS analysis.

**Additional file 2: Table S1.** General features of the complete genome of *Ruminiclostridium papyrosolvens* DSM2782.

**Additional file 3: Table S2.** List of the putative CAZymes in the genome of *R. papyrosolvens*.

**Additional file 4: Figure S2.** SDS-PAGE analysis of the secretomes isolated from the four growth conditions (glucose, cellobiose, cellulose and corn stover).

**Additional file 5: Figure S3.** Principle component analysis (PCA) of secretomes including three biological replicates under four carbon sources (glucose, cellobiose, cellulose and corn stover).

**Additional file 6: Table S3.** The LFQ intensity of proteins in secretomes under various carbon substrates in *R. papyrosolvens*.

**Additional file 7: Table S4.** List of signal-peptide-harboring proteins identified in each carbon source.

**Additional file 8: Table S5.** Relative expression level (Z-score) of expressed CAZymes under four carbon sources (glucose, cellobiose, cellulose and corn stover) with three biological replicates.

**Additional file 9: Figure S4.** Correlation of the expression level (Z-score) of 116 expressed CAZymes between cellobiose and corn stover.

**Additional file 10: Figure S5.** Elution effect of EDTA buffer for cellulose-binding proteins. Lanes 1 and 2 secreted proteins before and after incubated with cellulose; lanes 3–5 fractions washed from cellulose; lane 6 residual proteins binding to cellulose after 3 times of washing.

## Data Availability

The datasets generated during the current study are available in the Proteomics IDEntifications database (PRIDE) under Accession Number PXD013253.

## Abbreviations

CAZyme: carbohydrate-active enzymes
GH: glycoside hydrolase
PL: polysaccharide lyases
CE: carbohydrate esterase
GT: glycosyl transferase
AA: enzyme with auxiliary activities
CBM: carbohydrate-binding module
CBP: consolidated bioprocessing
LC–MS/MS: liquid chromatography–tandem mass spectrometry
PCA: principal component analysis
COG: cluster of orthologous group
SBP: solute-binding protein
ABC: ATP-binding cassette
CCR: carbon catabolite repression
CMC: carboxymethyl cellulose
SRPS: selective RNA processing and stabilization
TCS: two-component system
LFQ: label-free quantification
