# Supplementary figures and images for: Secretomic analyses of Ruminiclostridium papyrosolvens reveal its enzymatic basis for lignocellulose degradation
Source: Biotechnol Biofuels. 2019 Jul 15;12:183. doi: 10.1186/s13068-019-1522-8 (PMC6628489; doi:10.1186/s13068-019-1522-8)

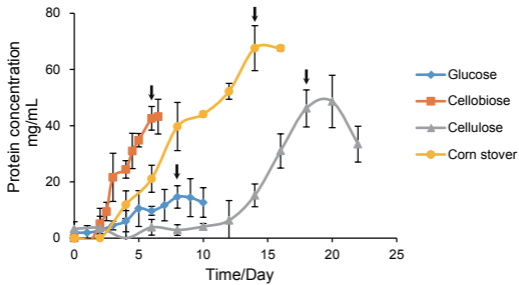

Supplement: Supplementary file 1 — Additional file 1: Figure S1. Growth curves of Ruminiclostridium papyrosolvens on glucose, cellobiose, cellulose and corn stover. Cell growth was monitored by determining the amount of proteins in supernatant of fermentation broths as described in Materials and Methods. The symbols indicate the means of three experiments, and the error bars indicate the standard deviations. The arrows show the time of sampling for MS analysis. [file 13068_2019_1522_MOESM1_ESM.pdf]

Cellobiose

Glucose

Cellulose

Corn stover

kDa

116.0

66.2

45.0

35.0

25.0

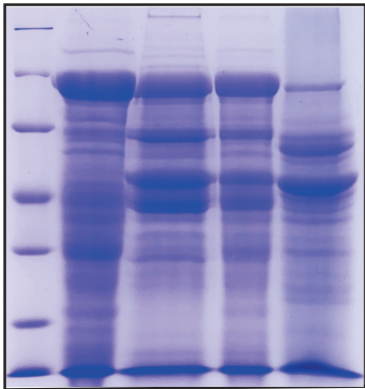

Supplement: Supplementary file 4 — Additional file 4: Figure S2. SDS-PAGE analysis of the secretomes isolated from the four growth conditions (glucose, cellobiose, cellulose and corn stover). [file 13068_2019_1522_MOESM4_ESM.pdf]

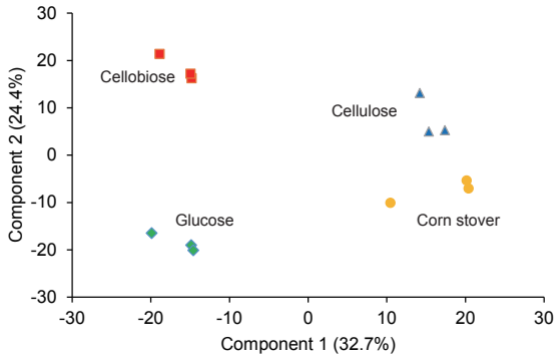

Supplement: Supplementary file 5 — Additional file 5: Figure S3. Principle component analysis (PCA) of secretomes including three biological replicates under four carbon sources (glucose, cellobiose, cellulose and corn stover). [file 13068_2019_1522_MOESM5_ESM.pdf]

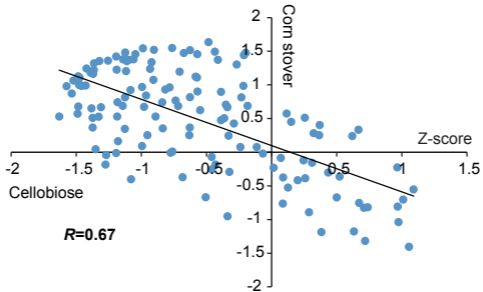

Supplement: Supplementary file 9 — Additional file 9: Figure S4. Correlation of the expression level (Z-score) of 116 expressed CAZymes between cellobiose and corn stover. [file 13068_2019_1522_MOESM9_ESM.pdf]

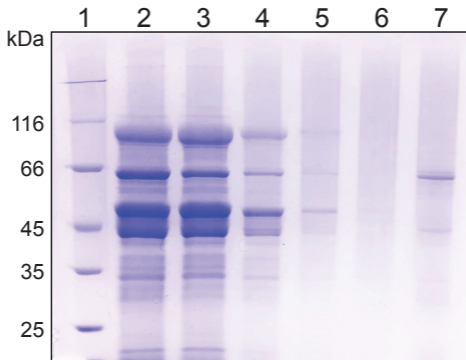

Supplement: Supplementary file 10 — Additional file 10: Figure S5. Elution effect of EDTA buffer for cellulose-binding proteins. Lanes 1 and 2 secreted proteins before and after incubated with cellulose; lanes 3–5 fractions washed from cellulose; lane 6 residual proteins binding to cellulose after 3 times of washing. [file 13068_2019_1522_MOESM10_ESM.pdf]
