# Supplementary material for: Secretomic analyses of Ruminiclostridium papyrosolvens reveal its enzymatic basis for lignocellulose degradation
Source: Biotechnol Biofuels. 2019 Jul 15;12:183. doi: 10.1186/s13068-019-1522-8 (PMC6628489; doi:10.1186/s13068-019-1522-8)
Supplement: Supplementary file 2 — Additional file 2: Table S1. General features of the complete genome of Ruminiclostridium papyrosolvens DSM2782. [file 13068_2019_1522_MOESM2_ESM.doc]

**Table S1. General features of the complete genome of *Ruminiclostridium papyrosolvens* DSM 2782**

| **Genome size (base pairs)** | 4,915,287 |
| --- | --- |
| **Contigs** | 31 |
| **G+C content (%)** | 37.0 |
| **Open reading frames** | 4193 |
| **Predicted protein encoding sequences** | 4039 |
| **Predicted sequences encoding RNA genes** | 79 |
| **rRNA** | 18 |
| **tRNA** | 57 |
| **CRISPRs loci** | 1 |
| **Non-coding RNAs** | 4 |
| **Pseudo genes** | 154 |
| **Insertion sequence (IS) elements** | 86^‡^ |
